# Thyroid Hormone Profile in Patients Ingesting Soft Gel Capsule or Liquid Levothyroxine Formulations with Breakfast

**DOI:** 10.1155/2016/9043450

**Published:** 2016-05-30

**Authors:** Carlo Cappelli, Ilenia Pirola, Elena Gandossi, Alessandra Cristiano, Linda Daffini, Barbara Agosti, Claudio Casella, Maurizio Castellano

**Affiliations:** ^1^Department of Medical and Surgical Sciences, Endocrine and Metabolic Unit, Clinica Medica, 2nd Medicina, University of Brescia, Spedali Civili di Brescia, 25123 Brescia, Italy; ^2^Department of Molecular and Translational Medicine, 3rd Division of General Surgery, University of Brescia, Spedali Civili di Brescia, 25123 Brescia, Italy

## Abstract

*Background*. Recently, it has been shown that liquid L-T4 formulation can be ingested with breakfast. This study looked to extend these findings by investigating whether a soft gel capsule formulation of L-T4 could also be ingested at breakfast time.* Methods*. 60 patients (18–65 yrs), previously submitted to thyroidectomy for proven benign goitre in stable euthyroidism receiving liquid L-T4 therapy ingested with breakfast, were enrolled. TSH, fT4, and fT3 levels were assessed in all the patients who were switched from liquid L-T4 to a soft gel capsule formulation at the same dosage of L-T4. After 6 months, TSH, fT4, and fT3 levels were determined again.* Results*. There were no differences in TSH levels, but fT3 and fT4 levels during treatment with the soft gel capsule were significantly lower than those at enrolment with the liquid L-T4 formulation (TSH median (min–max): 1.9 (0.5–4.0) versus 2.2 (0.5–4.5) mIU/L, fT3: 2.5 (2.4–3.1) versus 2.7 (2.4–3.3) pg/mL, *p* < 0.05, and fT4: 9.9 (8.0–13) versus 10.6 (8.6–13.8) pg/mL, *p* < 0.0001).* Conclusion*. Both liquid and soft gel formulations of L-T4 can be taken with breakfast. However, liquid L-T4 would be the preferred formulation for patients in whom even small changes in fT4 and fT3 levels are to be avoided.

## 1. Introduction

Levothyroxine (L-T4) is used worldwide as replacement therapy for patients with hypothyroidism, and for the last few years it has been the third most commonly dispensed therapy in the United States [1]. Synthetic L-T4 as a therapeutic agent was first used in the 1950s and was widely adopted as the primary agent for thyroid hormone replacement, replacing the natural desiccated thyroid extract that had been used over the previous 50-year period [2].

Although replacement therapy with levothyroxine has been prescribed for more than 60 years and is generally considered straightforward, cross-sectional surveys of patients on treatment with levothyroxine demonstrate that between 40% and 48% are either overtreated or undertreated [3, 4]. There are many potential explanations for this. Firstly, a number of factors may interfere with intestinal absorption of L-T4, including food, dietary fibre, coffee, drugs, gastric or intestinal resection, and disease [5]. As a result, current guidelines recommend that L-T4 is taken in a fasting state at least 30 minutes before breakfast [6–8].

Secondly, it is not always reliable to follow medical advice, especially for drug therapy [9]; this is especially relevant for L-T4 therapy as a significant number of patients have problems in postponing their breakfast by 30–60 minutes to ensure that they take L-T4 in the fasting state [10]. In the therapeutic scenario the recent introduction of nontablet L-T4 formulations, such as liquid and soft gel capsules, would seem to raise doubts as to this recommendation [5].

The results of a recent randomized, double-blind, placebo-controlled crossover trial showed that a liquid L-T4 formulation can be ingested during each patient's normal breakfast, mixed with tea, coffee, milk, cappuccino, orange juice, and so forth, thus potentially improving therapeutic compliance [11]. This study, the “TITI” study (Tirosint®-versus-Tiche® study, IBSA Italia), looked to extend these findings by investigating whether an L-T4 soft gel capsule formulation could also be safely and effectively administered to stable euthyroid patients during each patient's normal breakfast without any malabsorption.

## 2. Subjects and Methods

Eligible patients were selected by a search in the database for those treated and followed up at the Thyroid Unit of the Department of Clinical and Experimental Sciences, University of Brescia, Italy. Eligibility (search) criteria were as follows: (a) treatment of hypothyroidism with liquid L-T4 (IBSA Farmaceutici Italia Srl., Lodi, Italy) ingested during each patient's normal breakfast; (b) stable levothyroxine replacement over the last 6 months; (c) complete personal medical history; and (d) details of current drug therapy and any previous therapy. In order to avoid any possible confounders (i.e., the need to increase L-T4 therapy in patients affected by Hashimoto's thyroiditis during the study span) we enrolled only patients who had previously undergone total thyroidectomy for proven benign goitre.

All participants had to maintain the same breakfast habits and any ongoing therapy for the entire period of the study.

All patients were assessed for thyroid-stimulating hormone (TSH), free T4 (fT4), and free T3 (fT3) levels and were switched from liquid L-T4 to the soft gel capsule formulation at the same dosage of L-T4, ingested during each patient's normal breakfast. Serum concentrations of TSH, fT4, and fT3 were once again established after six months. All patients gave their informed consent to participate in the study, conducted in accordance with the Declaration of Helsinki. Institutional Review Board approval for this study was obtained.

Serum concentrations of fT4 (normal range 8.0–19.0 pg/mL, analytical sensitivity 1 pg/mL), fT3 (normal range 2.4–4.7, analytical sensitivity 0.35 pg/mL), and TSH (normal range 0.4–4.5 mIU/L, analytical sensitivity 0.004 mIU/L) were measured using a fully automated Architect i2000 analyzer (Abbott Diagnostics, Abbott Park, IL, USA) based on chemiluminescent magnetic immunoassay.

## 3. Statistical Analysis

Statistical analyses were performed using SPSS 17.0 software (SPSS, Inc., Evanston, IL, USA). Normal distribution was checked by the Shapiro-Wilk test. TSH, fT4, and fT3 levels distribution levels were nonnormally distributed and were not normalized by the usual procedures of data transformation; in these cases results are presented as median with minimum and maximum values. Comparisons between continuous variables were performed by paired samples *t*-test or related samples by the Wilcoxon signed rank test, as appropriate.

Two-tailed *p* < 0.05 was considered statistically significant.

## 4. Results

Of the 2371 assessed patients with hypothyroidism, 60 (51 females and 9 males, aged 47.7 ± 11.2 years) were eligible for the inclusion criteria and were enrolled for the study. All patients were euthyroid based on test results conducted 6 months before recruitment whilst receiving stable liquid L-T4 therapy ingested with breakfast (mean dosage 106.25 ± 24.28 *µ*g/day). A detailed description of breakfast composition, particularly related to insoluble fibres and/or soya milk, was obtained in each subject (Table 1).

The thyroid hormonal profiles during administration of liquid L-T4 six months before recruitment, at enrolment and after 6 months' treatment with L-T4 soft gel capsules, are shown in Table 2. There were no differences in TSH levels at the three different time points, but during treatment with the soft gel capsule formulation fT3 and fT4 levels were significantly lower than those found 6 months before recruitment and at enrolment with the liquid L-T4 formulation. No patients developed hypothyroidism during the period of the study.

A subgroup analysis was conducted on the last 30 consecutive patients using data after three months of treatment with L-T4 soft gel capsules (Table 3). There were no significant differences in hormone levels between 6 months before recruitment, enrolment, and at 3 months. Significant reductions in fT3 and fT4 levels were observed between 3 and 6 months' treatment with L-T4 soft gel capsules whereas there was a slight increase in TSH value.

## 5. Discussion

The main finding of this study is that both the liquid and soft gel capsule formulations of L-T4 can be taken with breakfast. Priority should however be given to the liquid L-T4 formulation in those patients where even a small change in fT4 and fT3 levels is to be avoided.

Current guidelines for the treatment of hypothyroidism recommend ingesting levothyroxine in the fasting state 60–30 minutes before breakfast or at bedtime, at least three hours after the evening meal, for optimal and consistent absorption [6–8]. This recommendation is based on study data demonstrating that concomitant ingestion of food [12–15], fibre [16], soya products [17], and coffee [13] is associated with higher serum TSH values in hypothyroid subjects treated with L-T4 as compared with the fasting state. Taking L-T4 with coffee, or with water followed by coffee within a few minutes, results in a poor TSH response in many patients [13]. The recent introduction of new nontablet formulations of L-T4, such as soft gel capsules and a liquid, could resolve this problem [5]. In one small study of hypothyroid patients, Vita et al. observed that the absorption of a soft gel preparation of L-T4 (Tiche capsules, IBSA, Switzerland) was not impaired by taking it with coffee [10]. The same authors also reported that the problem of incomplete absorption of L-T4 caused by proton pump inhibitor-induced increases in gastric pH was not observed with the L-T4 soft gel formulation [18]. Morelli et al. demonstrated that there is therapeutic equivalence in liquid L-T4 administration at breakfast or 10 min beforehand [19].

We have previously observed that taking L-T4 at 30 minutes before having breakfast was not associated with alterations in optimal TSH, fT4, and fT3 concentrations in a group of patients taking their dose of a liquid L-T4 formulation (Tirosint, IBSA, Italy) with coffee [20]. More recently, we clearly demonstrated in a randomized, placebo-controlled double-blind crossover trial that the thyroid hormone profiles are almost identical after the administration of the same dose of oral liquid L-T4 either with breakfast or in the fasting state (30 minutes before breakfast) [11]. This study confirms and extends these previous findings. To the best of our knowledge, no previous study has made comparisons between soft gel capsule and liquid formulations of L-T4 taken at breakfast time. The clinical relevance of the significant reduction in fT4 and fT3 serum levels after switching from liquid to soft gel capsule L-T4 is not clear. However, no patient developed hypothyroidism after 6 months of switching formulations. Nevertheless, further analysis of data on the last 30 patients would appear to indicate a progressive reduction in fT4 and fT3 levels after the switchover as time passed. At three months from switching over from liquid to soft gel capsule L-T4, TSH, fT4, and fT3 levels were superimposable on those found at recruitment, but reductions were seen over the next 3 months of therapy (i.e., by 6 months). The TSH had again slightly increased but the change was not of statistical significance. Based on these findings, it would be reasonable to assume that absorption of the soft gel capsule L-T4 formulation gradually diminishes as time passes when ingested with breakfast.

This study could be limited by the relatively short follow-up time. A longer follow-up during soft gel capsule treatment could yield further information on the trend of thyroid hormonal profile and the clinical significance of the hormonal changes demonstrated by this study. For this reason, large longitudinal prospective studies are needed to clarify this important issue.

In conclusion, the results of this study suggest that, in general, both the liquid and soft gel capsule formulations of L-T4 can be taken with breakfast. Priority should however be given to the liquid L-T4 formulation in those patients where even small changes in fT4 and fT3 levels need to be avoided, such as those receiving suppressive therapy for thyroid cancer and/or cardiopathic patients receiving substitutive L-T4 therapy.

## Figures and Tables

**Table 1:** Breakfast composition and concomitant drug treatment of the patients in the study.

Patient	Gender	Age	Breakfast	Concomitant drug(s) assumed at breakfast
1	F	54	Tea	Calcium
2	F	60	Milk	—
3	F	44	Tea	—
4	M	53	Coffee & yogurt	—
5	F	68	Coffee & biscuits & yogurt	—
6	M	55	Coffee & yogurt	—
7	F	60	Cappuccino	PPI
8	F	47	Cappuccino & biscuits	—
9	F	43	Coffee & biscuits	—
10	F	51	Cappuccino & fibres	—
11	M	40	Tea & biscuits	—
12	F	46	Coffee & fibres & yogurt	—
13	F	35	Coffee & milk & fibres	Iron
14	F	51	Cappuccino & orange juice	—
15	F	59	Coffee & milk & fibres	PPI
16	F	61	Tea & yogurt	—
17	M	51	Coffee & soya milk & fibres	Calcium
18	F	55	Coffee & yogurt	Calcium
19	F	44	Coffee & milk & fibres & biscuits	—
20	F	49	Coffee & biscuits	Calcium
21	F	27	Cappuccino & biscuits	—
22	F	36	Coffee & biscuits	—
23	F	55	Milk & biscuits & fibres	—
24	F	66	Orange juice	PPI
25	F	64	Fibres & fruits	—
26	F	69	Milk & biscuits	—
27	F	29	Orange juice	—
28	F	34	Cappuccino	—
29	F	55	Cappuccino & biscuits	—
30	F	59	Coffee & biscuits	—
31	F	46	Coffee	—
32	F	60	Coffee & biscuits	Calcium-PPI
33	M	53	Tea & biscuits & yogurt	—
34	F	40	Cappuccino & biscuits	—
35	F	24	Tea & biscuits	—
36	F	48	Coffee & milk & fibres	—
37	M	45	Coffee & biscuits	—
38	F	47	Coffee & fibres & yogurt	PPI
39	F	40	Coffee	—
40	F	30	Tea & biscuits	—
41	F	42	Milk	—
42	F	43	Milk & biscuits	—
43	F	36	Milk & biscuits	—
44	F	47	Cappuccino & biscuits	Calcium
45	F	53	Milk & fibres	—
46	F	56	Milk	—
47	F	24	Orange juice & biscuits & fibres	—
48	F	54	Coffee & biscuits	—
49	F	41	Tea & biscuits	—
50	F	41	Tea	—
51	F	49	Milk & fibres	—
52	F	43	Tea & fibres	—
53	F	25	Coffee	—
54	F	65	Coffee & biscuits	Calcium
55	M	65	Soya milk & biscuits	PPI
56	F	53	Cappuccino & biscuits	—
57	M	45	Tea	—
58	F	43	Coffee & biscuits	PPI
59	F	49	Cappuccino & biscuits	—
60	M	37	Tea & orange juice	—

PPI: proton pump inhibitors.

**Table 2:** Thyroid hormone levels six months before recruitment and at enrolment (both when taking liquid L-T4) and after 6 months' treatment with L-T4 soft gel capsules.

	6 months before recruitment	At enrolment	6 months after soft gel capsule
TSH (mIU/L)	1.9 (0.5–4.1)	1.9 (0.5–4.0)	2.2 (0.5–4.5)
fT4 (pg/mL)	10.5 (8.6–13.8)	10.6 (8.6–13.8)	9.9 (8.0–13)^*∗*^
fT3 (pg/mL)	2.7 (2.4–3.6)	2.7 (2.4–3.3)	2.5 (2.4–3.1)^+^

Median (min–max).

^*∗*^ *p* < 0.0001 versus 6 months before recruitment and at enrolment;  ^+^ *p* < 0.05 versus 6 months before recruitment and at enrolment.

**Table 3:** Thyroid hormone levels six months before recruitment and at enrolment (both when taking liquid L-T4) and after 3 and 6 months' treatment with L-T4 soft gel capsules (*n* = 30).

	6 months before recruitment	At enrolment	3 months after soft gel capsule	6 months after soft gel capsule
TSH (mIU/L)	1.9 (0.5–4.0)	1.9 (0.5–4.0)	2.0 (0.5–4.5)	2.2 (0.7–4.4)
fT4 (pg/mL)	10.5 (8.6–13.7)	10.5 (8.6–13.6)	10.4 (9.1–13)	9.9 (8.8–13.3)^*∗*^
fT3 (pg/mL)	2.8 (2.4–3.6)	2.7 (2.4–3.2)	2.7 (2.4–3.1)	2.5 (2.4–3.4)^*∗*^

Median (min–max).

^*∗*^ *p* < 0.05 versus at 3 months after soft gel capsule treatment, at enrolment, and before recruitment.

## References

[2] Lindholm J., Laurberg P. (2011). Hypothyroidism and thyroid substitution: historical aspects. *Journal of Thyroid Research*.

[3] Canaris G. J., Manowitz N. R., Mayor G., Ridgway E. C. (2000). The colorado thyroid disease prevalence study. *Archives of Internal Medicine*.

[4] Parle J. V., Franklyn J. A., Cross K. W., Jones S. R., Sheppard M. C. (1993). Thyroxine prescription in the community: serum thyroid stimulating hormone level assays as an indicator of undertreatment or overtreatment. *British Journal of General Practice*.

[5] Formenti A. M., Daffini L., Pirola I., Gandossi E., Cristiano A., Cappelli C. (2015). Liquid levothyroxine and its potential use. *Hormones*.

[6] Jonklaas J., Bianco A. C., Bauer A. J. (2014). Guidelines for the treatment of hypothyroidism: prepared by the American thyroid association task force on thyroid hormone replacement. *Thyroid*.

[7] Liwanpo L., Hershman J. M. (2009). Conditions and drugs interfering with thyroxine absorption. *Best Practice & Research: Clinical Endocrinology and Metabolism*.

[8] Garber J., Cobin R., Gharib H. (2012). Clinical practice guidelines for hypothyroidism in adults: cosponsored by the american association of clinical endocrinologists and the american thyroid association. *Endocrine Practice*.

[9] Düsing R., Lottermoser K., Mengden T. (2001). Compliance with drug therapy—new answers to an old question. *Nephrology Dialysis Transplantation*.

[10] Vita R., Saraceno G., Trimarchi F., Benvenga S. (2013). A novel formulation of L-thyroxine (L-T4) reduces the problem of L-T4 malabsorption by coffee observed with traditional tablet formulations. *Endocrine*.

[11] Cappelli C., Pirola I., Daffini L. (2016). A double-blind placebo-controlled trial of liquid thyroxine ingested at breakfast: results of the TICO study. *Thyroid*.

[12] Wenzel K. W., Kirschsieper H. E. (1977). Aspects of the absorption of oral L-thyroxine in normal man. *Metabolism*.

[13] Benvenga S., Bartolone L., Pappalardo M. A. (2008). Altered intestinal absorption of L-thyroxine caused by coffee. *Thyroid*.

[14] Bach-Huynh T.-G., Nayak B., Loh J., Soldin S., Jonklaas J. (2009). Timing of levothyroxine administration affects serum thyrotropin concentration. *Journal of Clinical Endocrinology and Metabolism*.

[15] Perez C. L. S., Araki F. S., Graf H., De Carvalho G. A. (2013). Serum thyrotropin levels following levothyroxine administration at breakfast. *Thyroid*.

[16] Liel Y., Harman-Boehm I., Shany S. (1996). Evidence for a clinically important adverse effect of fiber-enriched diet on the bioavailability of levothyroxine in adult hypothyroid patients. *Journal of Clinical Endocrinology and Metabolism*.

[17] Bell D. S. H., Ovalle F. (2001). Use of soy protein supplement and resultant need for increased dose of levothyroxine. *Endocrine Practice*.

[18] Vita R., Benvenga S. (2014). Tablet levothyroxine (L-T4) malabsorption induced by proton pump inhibitor: a problem that was solved by switching to L-T4 in soft gel capsule. *Endocrine Practice*.

[19] Morelli S., Reboldi G., Moretti S., Menicali E., Avenia N., Puxeddu E. (2015). Timing of breakfast does not influence therapeutic efficacy of liquid levothyroxine formulation. *Endocrine*.

[20] Cappelli C., Pirola I., Gandossi E., Formenti A., Castellano M. (2014). Oral liquid levothyroxine treatment at breakfast: a mistake?. *European Journal of Endocrinology*.

